# Delayed Versus Immediate Start of Chemotherapy in Asymptomatic Patients With Advanced Cancer: A Meta-Analysis

**DOI:** 10.1093/oncolo/oyad235

**Published:** 2023-08-17

**Authors:** Simone Augustinus, Gajanan Thurairajah, Marc G Besselink, Hanneke W M van Laarhoven, Martijn G H van Oijen, Tara M Mackay, Johanna W Wilmink

**Affiliations:** Department of Surgery, Amsterdam UMC, University of Amsterdam, Amsterdam, The Netherlands; Cancer Treatment and Quality of Life, Cancer Center Amsterdam, Amsterdam, The Netherlands; Cancer Treatment and Quality of Life, Cancer Center Amsterdam, Amsterdam, The Netherlands; Department of Medical Oncology, Amsterdam UMC, University of Amsterdam, Amsterdam, The Netherlands; Department of Surgery, Amsterdam UMC, University of Amsterdam, Amsterdam, The Netherlands; Cancer Treatment and Quality of Life, Cancer Center Amsterdam, Amsterdam, The Netherlands; Cancer Treatment and Quality of Life, Cancer Center Amsterdam, Amsterdam, The Netherlands; Department of Medical Oncology, Amsterdam UMC, University of Amsterdam, Amsterdam, The Netherlands; Cancer Treatment and Quality of Life, Cancer Center Amsterdam, Amsterdam, The Netherlands; Department of Medical Oncology, Amsterdam UMC, University of Amsterdam, Amsterdam, The Netherlands; Department of Surgery, Amsterdam UMC, University of Amsterdam, Amsterdam, The Netherlands; Cancer Treatment and Quality of Life, Cancer Center Amsterdam, Amsterdam, The Netherlands; Cancer Treatment and Quality of Life, Cancer Center Amsterdam, Amsterdam, The Netherlands; Department of Medical Oncology, Amsterdam UMC, University of Amsterdam, Amsterdam, The Netherlands

**Keywords:** meta-analysis, advanced cancer, asymptomatic, chemotherapy, timing

## Abstract

**Background:**

Due to increased use of imaging, advanced stages of cancer are increasingly being diagnosed in an early, asymptomatic phase. Traditionally, chemotherapy is started immediately in these patients. However, a strategy wherein chemotherapy is withheld until symptoms occur may be beneficial for patients in terms of quality of life (QOL). A systematic review regarding optimal timing of chemotherapy including survival and QOL is lacking.

**Methods:**

We systematically searched PubMed, EMBASE, and Cochrane for studies investigating the timing of start of chemotherapy in asymptomatic patients with advanced cancer. Overall survival (OS) was abstracted as primary, QOL, and toxicity as secondary outcomes. A meta-analysis was performed on OS. QOL was described using the global health status derived from the EORTC-QLQ-C30 questionnaire and toxicity as grade 3-4 adverse events.

**Results:**

Overall, 919 patients from 4 randomized controlled trials and 1 retrospective study were included. The included studies investigated colorectal cancer (*n* = 3), ovarian cancer (*n* = 1), and gastric cancer (*n* = 1). Pooled analysis demonstrated no significant differences in OS between delayed and immediate start of chemotherapy (pooled HR: 1.05, 95% CI, 0.90-1.22, *P* = .52). QOL, evaluated in 3 studies, suggested a better QOL in the delayed treatment group. Toxicity, evaluated in 2 studies, did not differ significantly between groups.

**Conclusion:**

This meta-analysis confirms the need for prospective studies on timing of start of chemotherapy in asymptomatic patients with advanced cancer. The limited evidence available suggests that delayed start of chemotherapy, once symptoms occur, as compared to immediate start in asymptomatic patients does not worsen OS while it may preserve QOL.

## Teaser

Cancer is increasingly being diagnosed in an early, asymptomatic phase. This review focuses on optimal timing of chemotherapy for asymptomatic patients with advanced cancer, weighing the benefits of early chemotherapy against the resulting toxicity and potentially lower quality of life.

**Implications for Practice**

Due to increased use of imaging, advanced stages of cancer are increasingly diagnosed in an early, asymptomatic phase. Traditionally, chemotherapy is started immediately in these patients; however, withholding chemotherapy until symptoms occur may be beneficial for patients in terms of quality of life (QOL). This meta-analysis included 919 asymptomatic patients with any type of advanced cancer (ie, colorectal cancer, ovarian cancer, and gastric cancer) from 4 randomized controlled trials and 1 retrospective study. Meta-analysis showed no statistically significant difference between delayed and immediate treatment in terms of overall survival. QOL was evaluated in 3 studies and suggested a better QOL for the delayed treatment group. This meta-analysis indicates that further prospective studies are needed to assess the merits of delayed start of systemic treatment in asymptomatic patients with advanced cancer.

## Introduction

Patients with advanced cancer palliative chemotherapy may improve both overall survival (OS) and quality of life (QOL) by decreasing tumor burden and thereby diminishing ­disease-related symptoms.^1,2^ However, in asymptomatic patients with advanced cancer, the benefits of starting chemotherapy must be carefully weighed against the toxicity and potentially lower QOL on the short term.

From clinical experience, the number of asymptomatic patients with advanced cancer seems to be increasing. This can partly be explained by an increase in standardized follow-up regimens after curative intent surgery to detect recurrence of disease at an early stage.^3,4^ For example, in pancreatic cancer, 97% of patients after pancreatic surgery undergo imaging during follow-up to detect potential recurrence. Of the patients with local or distant recurrence, 24% is asymptomatic at diagnosis.^3^ In addition, upcoming techniques such as FDG-PET (fluorodeoxyglucose positron emission tomography) and whole-body imaging can detect metastases in an early phase with increased accuracy.^5-7^ For example, in patients with breast cancer, whole body imaging causes an increase in diagnoses of metastatic disease in asymptomatic patients.^6,7^

As the number of asymptomatic patients with advanced cancer increases, it becomes more important to determine the optimal timing of start of chemotherapy. Immediate initiation of chemotherapy at diagnosis may improve OS. However, toxicity of chemotherapy may impair the QOL in asymptomatic patients, especially when the burden of disease remains low for a significant period of time. This systematic review and meta-analysis aims to study the effect of the timing of start of chemotherapy on OS and QOL in asymptomatic patients with advanced cancer.

## Materials and Methods

This systematic review and meta-analysis was performed according to the PRISMA guidelines.^8^

### Search Strategy

A systematic literature search was performed in PubMed, EMBASE (Ovid), and Cochrane library on January 26, 2022. The search included various synonyms for the words “metastatic,” “relapse,” “advanced,” “asymptomatic,” “time or delay,” and “survival.” The search for each database is depicted in Supplementary Table S1. Two authors (S.A. and G.T.) independently reviewed titles, abstracts, and full-texts for eligibility based on predefined inclusion and exclusion criteria. In case of different interpretation, consensus was reached in a consensus meeting with a third-party arbiter (J.W.).

### Study Eligibility

Studies were included if they evaluated the timing of chemotherapy in asymptomatic patients with advanced cancer. Any chemotherapy agent was allowed in any dosing schedule and method of administration. The use of targeted therapies or novel agents (eg, cetuximab) in addition to the chemotherapy treatment was also permitted. Advanced cancer was defined as metastatic, locally advanced, or recurrence of previous cancer. Randomized controlled trials (RCTs), prospective studies, and retrospective studies were included. Exclusion criteria were studies that investigated (neo)adjuvant chemotherapy or lymphomas. Also, trial protocols, conference abstracts, secondary publications of previously published studies, commentaries, and articles that were not available in full text were excluded, as well as publications in languages other than English.

### Data Collection

The following variables were extracted using a predefined data extraction file: publication details (ie, study title, authors, publication date, country, and study design), patient characteristics (ie, number of patients, sex, age, tumor stage, and site of primary tumor), and intervention characteristics (ie, type of chemotherapy, duration of treatment, start criteria of delayed treatment, and median follow-up). The primary outcome (ie, OS) and secondary outcomes (ie, QOL and toxicity) were extracted additionally.

QOL was evaluated using the European Organization for the Research and Treatment of Cancer Quality of Life Questionnaire C30 (EORTC-QLQ-C30).^9^ Outcomes of additionally used questionnaires were reported if they entailed different QOL items than the EORTC-QLQ-C30 items. EORTC-QLQ-C30 is a cancer-specific questionnaire and encompasses global health status, 5 functioning scales (ie, physical, role, emotional, cognitive, and social functioning), 8 symptom scales/items (ie, fatigue, nausea and vomiting, pain, dyspnea, insomnia, appetite loss, constipation, and diarrhea), and financial difficulties. All scales were reported separately if available. Authors were contacted for raw data on QOL outcomes; however, no data were received. Due to missing raw data of 3 out of 3 studies that reported on QOL, a ­meta-analysis was not possible, and descriptive analysis was performed. For toxicity grades 3-4, adverse events were evaluated according to the NCI CTCAE (National Cancer Institute Common Terminology Criteria for Adverse Events). Adverse events of toxicity grades 3-4 were reported and depicted as percentage per study and per group (ie, delayed vs. immediate).

### Quality Assessment

The quality of studies was assessed by 2 independent reviewers (S.A. and G.T.) using the Cochrane Risk-of-Bias 2 (RoB-2) tool for RCTs^10^ and the risk of bias in nonrandomized studies of interventions (ROBINS-I)^11^ tool for retrospective cohort studies. Any disagreements were resolved in a consensus meeting with a third-party arbiter (J.W.)

### Statistical Analysis

Baseline characteristics were described per study. Characteristics were summarized as frequencies with proportions for binary or categorical variables, or as mean with SD or as median with (interquartile) range (IQR) for continuous variables as appropriate. The software program Digitizelt was used to read the coordinates of the Kaplan-Meier curve when the median OS was not mentioned.^12^

Meta-analysis was performed using Cochrane Review Manager version 5.4. For this purpose, studies that compared delayed chemotherapy versus immediate chemotherapy were included. The hazard ratios (HR) reported in the studies were used. When HRs were not explicitly mentioned in the articles, they were calculated with combinations of minimal of 2 of the following variables available in the articles: (log)HR, SE, variance, (log)confidence interval, *Z*-value, or *P*-value. When these variables were not available, they were calculated through the calculation methods of Tierney et al.^13^ Two sensitivity analysis were performed: exclusion of the retrospective article^14^ and exclusion of the article with the largest population (compromising more than half of study population).^15^ Because of heterogeneity in study populations and included tumor types, random effects instead of fixed effects were used. A *P*-value of below .05 was considered statistically significant.

## Results

### Search Results

**Figure 1.**
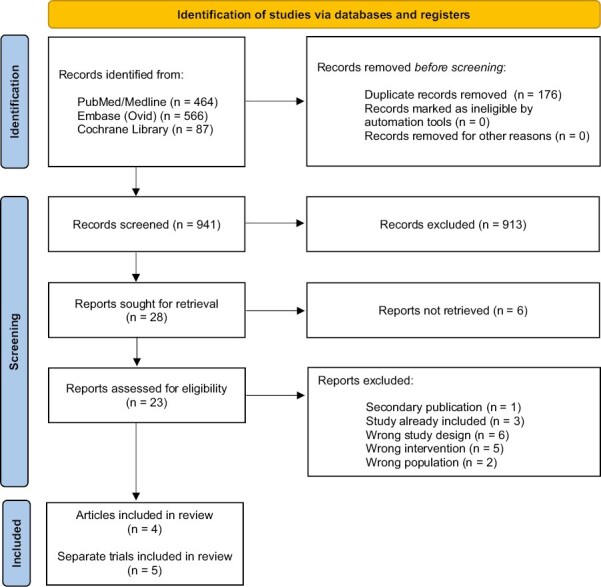
PRIMSA flow diagram.

A total of 1117 studies were identified. After title and abstract screening, 28 studies were included for full text screening. Six studies were excluded because no full-texts were available, but only conference abstracts. After full text screening of 23 studies, 4 could be included in this systematic review. One study (ie, Ackland et al^16^), included the results of 2 studies, yet were published as one joint paper in the form of a meta-analysis. We extracted and reported relevant data from both studies separately. We defined the studies as “Ackland 2005a” for the trial in Australia and New Zealand and “Ackland 2005b” for the Canadian trial. Hereby, the total number of included studies comes to 5. The PRISMA flow diagram in Fig. 1 displays the study selection.

### Study Characteristics

The 5 included studies consisted of 4 RCTs^15-17^ and 1 retrospective study^17^ (Table 1). In all studies, the timing of chemotherapy was investigated using 2 treatment arms: delayed treatment group and an immediate treatment group. The studies compromised a total of 919 patients, of which the largest study contained 529 patients (57.6%). In total, 467 patients (50.8%) received delayed treatment. Three studies included patients with colorectal cancer,^16,17^ one with ovarian cancer,^15^ and one with gastric cancer.^14^ In 3 out of 4 RCTs, patients were randomized when diagnosis of advanced cancer was confirmed.^16,17^ In the other RCT, the patients with ovarian cancer were randomized when the CA-125 concentration was elevated to twice the upper limit of normal during follow-up after primary chemotherapy.^15^ The start of delayed treatment varied amongstudies. Delayed treatment was started when (predefined) symptoms occurred in 3 out of 5 studies.^16,17^ In one study after clinical recurrence,^15^ and in another a minimum of 4 weeks after diagnosis (regardless of whether symptoms had occurred).^14^ In all of the studies, patients in the delayed treatment group received less chemotherapy (range 57%-88%) compared to the immediate treatment group. The median follow-up of all studies varied between 11 and 60 months. All studies used OS as primary outcome. Secondary outcomes used can be seen in Supplementary Table S2.

**Table 1.** Summary of the characteristics of the included studies.

Study	Tumor type	Tumor/treatment stage	Country	Study design	Total number of patients	Median age in years^*^	Start criteria of delayed treatment	Percentage patients received chemotherapy (immediate, delayed)	Type of chemotherapy	Median follow-up^**^
Ackland et al^16^	Colorectal cancer (CRC)	Local recurrence or metastases (not amenable curative treatment) in first-line therapy setting	Australia	RCT	101	65.3 (IQR 36-77)	Development of predefined symptoms	98%, 65%	Mayo clinic schedule^a^ or weekly 5-FU + LV	55 months
Ackland et al^16^		Local recurrence or metastases (not amenable curative treatment) in first-line therapy setting	Canada	RCT	67	63.7 (IQR 50-78)	Development of predefined symptoms	100%, 78%	Mayo clinic schedule^a^	55 months
Glimelius ^17^		Noncurable metastatic cancer in first-line therapy setting	Sweden and Norway	RCT	183	60.0^b^ (IQR 35-75)	Development of symptoms	89%, 57%	Direct treatment: MFL regimen^c^. Delayed treatment: MFL or a clinical chemotherapy trial^d^	NR (minimum of 12 months)
Elimova et al^14^	Gastric cancer	Stage IV in first-line therapy setting	US	Retrospective	39	60.0^e^ (IQR 33-83)	After minimal of 4 weeks after diagnosis	NR	Platinum analog + antimetabolite, or platinum analog antimetabolite + taxane, or other (not specified)	11 months (IQR 0.5-130.6)
Rustin et al^15^	Ovarian cancer	Relapsed ovarian cancer (>2× Ca-125 after initial complete remission of ­platinum-based chemotherapy)	UK, Spain, Norway, the Netherlands, France, Russia, Belgium, Ireland, Austria, and South Africa	RCT	529	61.0 (IQR 53-68)	Development of clinical recurrence	96%, 88%^f^	Choice of chemotherapy according to standard local practice	60 months (IQR 37.4-81.8)

*Delayed group.

**On primary outcome (survival).

^a^Mayo-clinic schedule: 5-FU and LV on day 1-5 repeated every 28 days.

^b^Mean instead of median.

^c^Methotrexate, followed by 5-FU, and LV.

^d^Comparing MFL with 5-FU, MFL with 5-FU and LV, or pilot study changing intervals of 5-FU and LV.

^e^Median age of all patients, as median age within delayed arm was not reported.

^f^Percentage patients starting second-line chemotherapy used, as the first-line chemotherapy included randomization errors. Abbreviations: RCT: randomized controlled trial; NR: not reported; 5-FU: 5-fluorouracil; LV: leucovorin; MFL: methotrexate, 5-fluororacil, leucovorin; FU: fluorouracil.

### Risk of Bias

Of the 4 RCTs, 1 study had a low overall risk of bias,^15^ and 3 studies had some concerns (Supplementary Table S3A).^16,17^ Moderate risk of bias was mostly due to the randomization process. The retrospective study was classified as moderate risk of bias (Supplementary Table S3B).^14^

### Overall Survival

**Figure 2.**
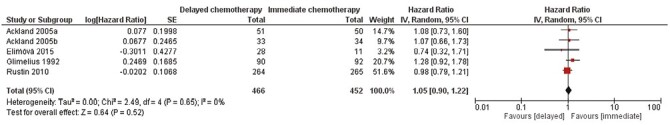
Meta-analysis of overall survival in patients with advanced cancer comparing delayed and immediate chemotherapy.

The median OS in the delayed treatment group varied between 9.0 and 27.1 months, and in the immediate group between 11.9 and 25.7 months (Table 2). All of the included studies could be included in the meta-analysis.^14-17^ The ­meta-analysis found no significant differences in terms of OS comparing delayed and immediate treatment (pooled HR 1.05, 95% CI, 0.90-1.22, *P* = .52, Fig. 2). A sensitivity analysis including only the RCTs showed comparable results (pooled HR 1.06, 95% CI, 0.91-1.23, *P* = .46, Supplementary Fig. S1). A sensitivity analysis excluding the article with the largest population, compromising more than half of patients, showed comparable results as well (HR 1.01, 95% CI, 0.81-1.25, *P* = .94, Supplementary Fig. S2).^15^

**Table 2.** The median OS and the total number of patients per intervention arm of each study.

Study	Type of cancer	Median OS in months	
		Immediate treatment	Delayed treatment
Ackland et al^16^	Colorectal cancer	15.5	11.9
Ackland et al^16^		11.9	10.2
Glimelius^17^		14.0	9.0
Elimova et al^14^	Gastric cancer	13.8^a^	16.7^a^
Rustin et al^15^	Ovarian cancer	25.7	27.1

When not specified, OS was defined as the date of randomization to death or last follow-up.

^a^OS was defined as the date of therapy initiation to death or last follow-up.

Abbreviation: OS: overall survival.

### Quality of Life

Three studies examined QOL (*N* = 697).^.15,16^ In 2 studies including patients with colorectal cancer, QOL was evaluated every 2 months.^16^ In both these studies, global health status in the delayed treatment group was higher at all time points compared to the immediate treatment group, except at 8 months when QOL was the same. However, these differences were not statistically significantly different, which was also the case for all separate QOL domains. In the study evaluating patients with ovarian cancer, QOL was measured before each chemotherapy cycle until the end of treatment.^15^ The median time spent in good global health score (defined as improved or no more than 10% decrease from prerandomization score) was higher in the delayed treatment group (9.2 months, 95% CI, 6.4-10.5) compared to the immediate treatment group (7.2 months, 95% CI, 5.3-9.3). Time from randomization to first deterioration in global health score or death (defined as more than 10% decrease from prerandomization score or death) was significantly higher in the delayed treatment group compared to the immediate treatment group (5.8 vs. 3.2 months, *P* = .002). Subgroup analysis of individual components of the QOL subscales, indicated faster deterioration in global health score in the immediate treatment group for almost all subscales, this was significant for the subscale: role, emotional, social, and fatigue.

### Toxicity

Three RCTs in patients with colorectal cancer reported adverse events (*N* = 222).^16,17^ Two RCTs compared grades 3-4 toxicity between the delayed and immediate treatment group.^16^ The total percentage of grades 3-4 adverse events in the whole group was not reported, only the incidence of specific (predefined) grades 3-4 adverse events. There were no statistically significant differences between the 2 groups, but the incidence of specific grades 3-4 events varied between 0% and 46% in the delayed treatment group and between 0% and 29% in the immediate treatment group (Table 3). In the third RCT, only the toxicity in the immediate treatment group was evaluated, and the incidence of specific grades 3-4 events varied between 0% and 4% (Table 3).^17^ In this RCT, the total percentage of grades 3-4 adverse events was reported, and only 4/82 patients (4.9%) reported grade 3 and 4 toxicity.

**Table 3.** The number of reported events of different types of grade 3 or 4 toxicities per study.

Type of toxicity *n*(%)	Ackland 2005a^16^		Ackland 2005b^16^		Glimelius 1992^17^
	Immediate treatment	Delayed treatment	Immediate treatment	Delayed treatment	Immediate treatment
	*n* = 49	*n* = 31	*n* = 34	*n* = 26	*n* = 82
Alkaline phosphatase	1 (2%)	7 (23%)	0	1 (4%)	NR
Aspartate aminotransferase	0	0	0	0	NR
Bilirubin	1 (2%)	2 (6%)	0	5 (19%)	NR
Conjunctivitis	NR	NR	NR	NR	1 (1%)
Creatinine/renal failure	0	0	1 (3%)	NR	2 (2%)
Dermatitis	0	0	0	1 (4%)	0
Diarrhea	10 (20%)	5 (16%)	9 (26%)	4 (15%)	3 (4%)
Hemoglobin	0	1 (3%)	0	2 (8%)	NR
Infection	4 (8%)	0	3 (9%)	2 (8%)	NR
Lactate dehydrogenase	7 (14%)	7 (23%)	NR	NR	NR
Leukopenia	0	0	3 (9%)	5 (19%)	2 (2%)
Nausea and vomiting	3 (6%)	5 (16%)	6 (18%)	0	1 (1%)
Neutrophils	4 (8%)	1 (3%)	8 (24%)	6 (23%)	NR
Other	3 (6%)	2 (6%)	10 (29%)	12 (46%)	0
Platelets	0	1 (3%)	0	0	2 (2%)
Stomatitis	1 (2%)	4 (13%)	9 (26%)	4 (15%)	1 (1%)

Analysis in patients that received (at least one cycle) of chemotherapy.

Abbreviation: NR: not reported.

## Discussion

This systematic review and meta-analysis on the optimal timing of palliative chemotherapy in 919 asymptomatic patients with advanced cancer from 4 RCTs and 1 retrospective cohort study (ie, colorectal cancer, ovarian cancer, and gastric cancer) showed no statistically significant difference between immediate and delayed treatment in terms of OS. QOL was evaluated in 3 out of 5 studies and suggested a better QOL of patients in the delayed treatment group.

Only one other (previously performed) systematic review assessed the timing of start of chemotherapy in asymptomatic patients with advanced cancer. This 2018 review focused on patients with colorectal cancer only, and included 3 RCTs, which are also included in our analysis.^18^ Compared to this previous review, our study included almost a doubled number of patients, as one retrospective study on gastric cancer (*n* = 39) and an RCT on ovarian cancer (*n* = 264), were also included. Pooled survival outcomes in both studies are comparable (previous review,^18^ (*n* = 175) HR 1.17, 95% CI, 0.93-1.46; current study, (*n* = 452): HR 1.05, 95% CI, 0.90-1.22). It is somewhat concerning that only 2 additional studies have been published on this topic since the previous review. This illustrates the limited evidence available. On the contrary, for other types of systemic treatment, such as immunotherapy, targeted therapy, and hormonal therapy, more studies on timing of treatment of asymptomatic advanced cancer have been performed. In patients with asymptomatic metastatic renal cell cancer, 3 studies indicate a positive role of delayed treatment with immunotherapy or targeted therapy in terms of safety, OS, and progression-free survival.^19-21^ In addition, a review including 8 studies with locally advanced or asymptomatic metastatic prostate cancer showed a nonsignificant trend in favor of early hormonal treatment in terms of OS (HR 1.23, 95% CI, 0.88-1.71).^22^

Multiple types of cancer are included in this review. It can be debated whether these results can be pooled, as every type of cancer is unique, given the inequalities in prognosis, treatment strategies, follow-up schedules, and effect of treatments on QOL. For example, metastatic gastric cancer has the lowest percentage of 5-year survival (6%), followed by metastatic colorectal cancer (14%) and metastatic ovarian cancer (30%).^23-25^ However, by presenting the results in a relative measure, namely as HRs, survival outcomes are standardized. To take the heterogeneity of the different types of cancer into account, random effects instead of fixed effects were used in the analysis.

In this review, most studies included only a small number of patients. Five of the 6 studies included less than 100 patients, and one trial included almost half of the patients.^15^ The fact that one trial included almost half of the patients, however, did not influence the results as shown by our sensitivity analysis. The limited number of included patients can be explained by difficulties in recruitment, as 3 out of 4 included RCTs reported recruitment issues.^15,16^ In the 2 most recent RCTs on patients with colorectal cancer,^16^ due to slow accrual, recruitment was suspended before the sample size was reached. In the RCT including patients with ovarian cancer,^15^ the trial management group considered trial closure options after the study had been open for longer than 10 years. This could possibly be due to a strong preference of patients for one of the treatment arms. Therefore, in future studies on the timing of start of chemotherapy of asymptomatic advanced cancer, recruitment problems should be taken into account in the design of the trials. It can be suggested to use a Trials within Cohorts (TwiCs) design, which aims to reduce recruitment difficulties and disappointment bias in pragmatic trials.^26^ In this design, on cohort enrollment, broad informed consent for randomization is asked, after which cohort participants can be randomized to interventions and asked for additional informed consent or serve as controls without further notification.^26^ Besides improving recruitment, this design also allows evaluation of patients acceptability of the intervention (eg, delayed chemotherapy) in clinical practice.^27^

As this review shows no difference between immediate and delayed treatment groups in terms of OS, the effect on QOL is expected to become more important in clinical decision making. This is underscored by Meropol et al who found that 55% of patients with advanced cancer valued QOL and survival equally, while 27% valued QOL over survival.^28^ Our results suggest that there may be a benefit in QOL for the delayed treatment group; however, some limitations have to be taken into account. First, of the included studies, QOL was only evaluated in 3 out of 5 studies and could not be evaluated in a meta-analysis due to different time points measured in each trial. Second, in the studies that evaluated QOL, the treatment allocation could not be blinded, and therefore, the outcomes reported could be affected by bias. A benefit in QOL for the delayed treatment group is in line with our expectations, as asymptomatic patients initially have no complaints, and therefore, any kind of toxicity of chemotherapy can impair QOL. Interestingly, in the 2 RCTs evaluating patients with colorectal cancer, no significant differences in grade 3 or 4 adverse events were seen comparing the 2 treatment groups.^16^ However, these studies were not powered to show differences in QOL or toxicity. Thereby, when taking a closer look at the evaluation of toxicity of patients receiving delayed treatment in both these RCTs, toxicity was only measured in the patients that actually received chemotherapy. Namely, only 31/51 patients (60.8%) in the Australian trial and 26/33 patients (78.8%) in the Canadian trial, respectively. This might bias the results, as less toxicity might be present in the delayed treatment group when taking in account the all patients.

Thereby, in most types of cancer (regardless of symptoms), a clear association is seen with palliative chemotherapy and an improved OS and QOL.^1,2^ Therefore, monitoring symptoms and physical status seems critical when deciding to delay treatment, as it is possible that due to delayed treatment patients miss the window of opportunity to start chemotherapy when symptoms develop. In this review, even though no difference in survival was seen, patients in the delayed treatment group received less chemotherapy compared to patients in the immediate treatment group within all trials (Table 1). Therefore, to adequately inform patients about the choice between delayed and immediate therapy, combined measurements such as quality-adjusted life years (QALYs) could be used.^29^ QALYs are calculated by using the QOL scores of the EQ-5D questionnaire, combined with survival data.^30^ As QALYs combine both morbidity and mortality in a single metric, it provides an unique way to inform the patient about the actual consequences in daily practice.^29^ In addition, using QALYs enables the researcher to calculate the ­cost-ffectiveness when necessary.

The results of this systematic review should be interpreted in light of some limitations. First, only studies on 3 types of cancer are included. The results should, therefore, be interpreted with caution and may not be representative for all types of cancer. It could be considered to also add the studies including other types of systemic treatment (eg, hormonal and target treatment) to increase generalizability. However, this would also decrease the validity of our research question. Second, some studies are somewhat older and contain dated treatments effecting the results. For example, for colorectal cancer, the chemotherapy regimens used in the trials included are now anachronistic, as the triple regimen FOLFOXIRI (fluorouracil, oxaliplatin, and irinotecan) in combination with EGFR and VEGFR inhibitors have dramatically changed the prognosis of this disease.^31^ Third, all studies were of limited quality, as 3 out of 4 RCTs showed some concerns in risk of bias assessment, and the retrospective study had a moderate risk of bias. Nevertheless, this systematic review provides an overview of the evidence available on the timing of treatment asymptomatic patients with various types of advanced cancer.

## Conclusion

Limited evidence exists on timing of start of chemotherapy for asymptomatic patients with advanced cancer. In these studies, delayed administration of chemotherapy does not result in worse OS compared to immediate treatment and may result in better QOL. The option of delayed start of systemic therapy should be discussed with asymptomatic patients within the process of shared-decision making; however, additional research should be performed within specific cancer types to adequately inform patients.

## Supplementary Material

oyad235_suppl_Supplementary_Figures

oyad235_suppl_Supplementary_Tables

## Data Availability

No new data were generated or analyzed in support of this research. Data can be made available upon reasonable request.
