# Supplementary material for: Delayed Versus Immediate Start of Chemotherapy in Asymptomatic Patients With Advanced Cancer: A Meta-Analysis
Source: Oncologist. 2023 Aug 17;28(11):961–8. doi: 10.1093/oncolo/oyad235 (PMC10628561; doi:10.1093/oncolo/oyad235)

**
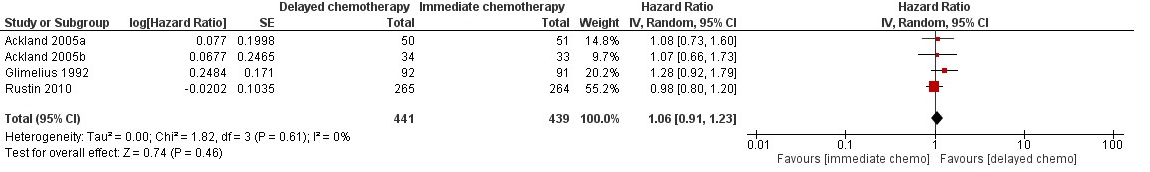
SUPPLEMENTARY FIGURE S1.** Sensitivity analysis of meta-analysis OS including only RCTs

RCTs = randomized controlled trials

**SUPPLEMENTARY FIGURE S2**. S Sensitivity analysis of meta-analysis OS excluding the article with the largest population (Rustin^15^)


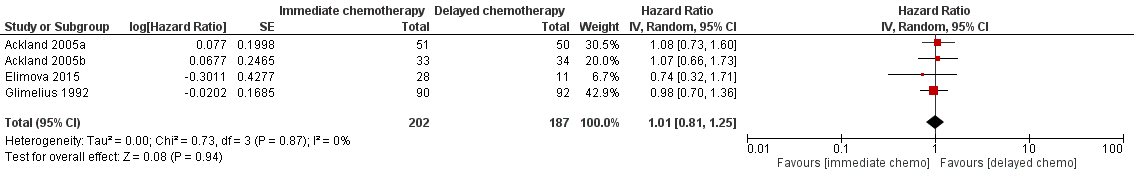

Supplement: oyad235_suppl_Supplementary_Figures [file oyad235_suppl_supplementary_figures.docx]
