# Supplementary material for: Delayed Versus Immediate Start of Chemotherapy in Asymptomatic Patients With Advanced Cancer: A Meta-Analysis
Source: Oncologist. 2023 Aug 17;28(11):961–8. doi: 10.1093/oncolo/oyad235 (PMC10628561; doi:10.1093/oncolo/oyad235)
Supplement: oyad235_suppl_Supplementary_Tables [file oyad235_suppl_supplementary_tables.docx]

**SUPPLEMENTARY TABLE S1**. Search strategy

| **Database** | **Search** | **Results** |
| --- | --- | --- |
| **Pubmed** | ("Neoplasm Metastasis"[Mesh] OR metasta*[tiab] OR ((advanced[tiab] OR relapse*[tiab]) AND (cancer[tiab] OR neoplasm*[tiab] OR lymphoma*[tiab]))) AND ("Time-to-Treatment"[Mesh] OR "Time Factors"[Mesh] OR "Watchful Waiting"[Mesh] OR wait time*[tiab] OR waiting time*[tiab] OR time to treatment[tiab] OR treatment delay*[tiab] OR delayed treatment[tiab] OR therapy delay*[tiab] OR delayed therap*[tiab] OR early treatment[tiab] OR watch and wait[tiab] OR watchful waiting[tiab]) AND ("Survival Rate"[Mesh] OR "Survival Analysis"[Mesh] OR "Survival"[Mesh] OR "mortality" [Subheading] OR survival[tiab] OR mortalit*[tiab]) AND (asymptomatic*[tiab] OR silent[tiab] OR occult[tiab] OR symptomless[tiab] OR symptom-free[tiab] OR before clinical relapse*[tiab] OR early treatment of relapse[tiab] OR prolonged time to treatment initiation*[tiab]) | 464 |
| **EMBASE** | #1 exp metastasis/ or advanced cancer/ or   metasta*.ti,ab,kw.  #2 ((advanced or relapse*) adj6 (cancer or neoplasm* or   lymphoma*)).ti,ab,kw .  #3 1 or 2  #4 time to treatment/ or time factor/ or watchful   waiting/  #5 (wait* adj3 time).ti,ab,kw.  #6 (time adj3 (treatment* or therap*)).ti,ab,kw.  #7 ((time or delay* or early) adj3 (treatment* or   therap*)).ti,ab,kw.  #8 (watch* adj3 wait*).ti,ab,kw.  #9 4 or 5 or 6 or 7 or 8  #10 survival rate/ or survival analysis/ or exp survival/ or   exp mortality/ or (survival or mortalit*).ti,ab,kw.  #11 asymptomatic disease/ or (asymptomatic* or silent or   occult or symptomless or before clinical relapse* or   early treatment of relapse or prolonged time to   treatment initiation*).ti,ab,kw.  #12 (symptom* adj3 free).ti,ab,kw.  #13 11 or 12  #14 3 and 9 and 10 and 13 | 566 |
| **Cochrane Libarary** | #1 (metasta*):ti,ab,kw  #2 ((advanced or relapse*) near/6 (cancer or neoplasm*   or lymphoma*)):ti,ab,kw  #3 #1 or #2 64013  #4 (wait* near/3 time):ti,ab,kw  #5 (time near/3 (treatment* or therap*)):ti,ab,kw  #6 ((time or delay* or early) near/3 (treatment* or  therap*)):ti,ab,kw  #7 (watch* near/3 wait*):ti,ab,kw  #8 #4 or #5 or #6 or #7  #9 (surviv* or mortalit*):ti,ab,kw  #10 (asymptomatic* or silent or occult or symptomless or "before clinical relapse*" or "early treatment of relapse" or "prolonged time to treatment initiation*"):ti,ab,kw  #11 symptom* near/3 free  #12 #10 or #11  #13 #3 and #8 and #9 and #12 | 87 |
| **Total** |  | 1117 |

SUPPLEMENTARY TABLE S2. Outcome measurements

| ***Study*** | ***Primary Outcome*** | ***Secondary outcomes*** |
| --- | --- | --- |
| **Ackland 2005a**^16^ | Overall survival | Time to disease progression  Quality of life (overall and individual domains)  Adverse events |
| **Ackland 2005b**^16^ | Overall survival | Time to disease progression  Quality of life (overall and individual domains)  Adverse events |
| **Glimelius 1992**^17^ | Overall survival | Median symptom-free survival  Progression free survival  Adverse events |
| **Elimova 2015**^14^ | Overall survival | NA |
| **Rustin 2010**^15^ | Overall survival | Time to second line chemotherapy  Time to third line treatment or death (whichever occurred first)  Duration of good quality of life in the global health score  Time of first global health detoration |

NA = Not applicable

**SUPPLEMENTARY TABLE S3a**. Risk of bias in RCTs

| **Study** | **Domain 1.** Randomization process | **Domain 2.** Deviations from intended interventions | **Domain 3.**  Missing outcome | **Domain 4.** Measurement of outcome | **Domain 5.** Selection of the reported results | **Overall.** Risk of bias |
| --- | --- | --- | --- | --- | --- | --- |
| Ackland 2005a^20^ |  |  |  |  |  |  |
| Ackland 2005b^20^ |  |  |  |  |  |  |
| Glimelius 1992^22^ |  |  |  |  |  |  |
| Rustin 2010^19^ |  |  |  |  |  |  |

RCTs: randomized controlled trials. Green: low risk of bias. Yellow: some concerns. Red: high risk of bias.

**TABLE S3b**. Risk of bias in retrospective studies

| **Study** | **Domain 1.**  Confouding | **Domain 2.**  Selection of participants | **Domain 3.**  Classification of interventions | **Domain 4.**  Deviations from intended interventions | **Domain 5.**  Missing data | **Domain 6.** Measurement of outcome | **Domain 7.** Selection of the reported result | **Overall.** Risk of bias |
| --- | --- | --- | --- | --- | --- | --- | --- | --- |
| Elimova 2015^18^ |  |  |  |  |  |  |  |  |

Green: low risk of bias. Yellow: some concerns.
